# Supplementary material for: How Has the COVID-19 Pandemic Affected the Way We Access and Interact with the Countryside and the Animals within It?
Source: Animals (Basel). 2021 Aug 2;11(8):2281. doi: 10.3390/ani11082281 (PMC8388493; doi:10.3390/ani11082281)
Supplement: Supplementary file 1 [file animals-11-02281-s001.zip › animals-1307656-supplementary.pdf]

How has the COVID-19 pandemic affected the way we access and interact with the countryside?

Page 1: Participant Information and Consent

The impact of COVID-19 on people's behaviour regarding exercise and activities in the countryside.

I am a masters by research student studying in the at the University of Bristol. I am conducting research into whether since the COVID-19 there has been an increase in people walking in and interacting with the countryside.

If you ever take walks in the countryside/green spaces I would be very grateful if you could spare about **5 minutes** to take part in my study by completing this questionnaire. In the context of this questionnaire **countryside** refers to any **general green areas** such as such as **fields, parks, woodlands, forests** etc. but **not** your private back garden.

You must be **at least 18** to take part and live in the UK.

Why is this research important?

It is important to quantify how the COVID-19 lockdown has had an impact on human behaviour, so changes in how people experience and interact with the countryside can be better understood.

Do I have to take part?

No, this questionnaire is entirely voluntary. Once you have started you are also under no

obligation to complete the questionnaire and may stop at any time without a reason.

What will happen to me if I take part and what will I have to do?

- You will answer the questions in this survey, you may leave out any questions you do not wish to respond to
- There are no right or wrong answers so please just respond as honestly as you can
- You must press the **'Finish'** button in order for your responses to be submitted

What are the possible disadvantages and risks of taking part?

There are no risks associated with taking part in this study.

Will my taking part in this study be kept confidential?

- All the answers you provide will remain anonymous
- However, as your data is anonymous you will not be able to withdraw it from the study once you have completed the questionnaire

What will happen to the results of the research study?

The aim is to publish the results in an academic journal alongside the findings from another questionnaire. A short summary will be made available on social media which participants and members of the public can view.

Who has reviewed the study?

This study has received ethical approval from the University of Bristol Faculty of Health Sciences Research Ethics Committee.

Further information and contact details

If you have any questions or concerns, please email me: amelia.cameron@bristol.ac.uk

or my supervisor: jo.hockenhull@bristol.ac.uk

1. I agree to participate under the conditions listed above: ✱ *Required*

◯ Yes

Page 2: Background Information

2. Where do you live?

- South East England
- South West England
- East Midlands
- West Midlands
- East of England
- North East England
- North West England
- Wales
- Scotland
- Northern Ireland

3. How would you best describe where you live?

- City
- Inner City
- Suburb/Town
- Village
- Rural

4. Do you have access to any of the following either at or near your home? ('**Near**' refers to **close enough** that you could **access** this place easily as part of your **local daily permitted exercise** when UK **lockdown** was **first enforced**, before you were permitted to drive unlimited distances for exercise.) (select all that apply)

- A private garden just for your household

- [ ] A shared garden, for your and other households in the same building/nearby
- [ ] A balcony/roof terrace
- [ ] An Allotment
- [ ] Privately owned land e.g. fields, woodlands
- [ ] Access to other private outdoor area
- [ ] No access to any form of private or shared private outdoor spaces

4.a. If you selected Access to other private outdoor area, please specify:

5. How old are you in years?

- ○ 18-24
- ○ 25-34
- ○ 35-44
- ○ 45-54
- ○ 55-64
- ○ 65-74
- ○ 75-84
- ○ 85+

Page 3: Lockdown and Use of Green Spaces

6. Before the UK government announced the COVID-19 lockdown, how often did you usually go for walks or spend time in in the countryside/green spaces?

- At least once/day
- 4-6 times/week
- 2-3 times/week
- Once/week
- Once every two weeks
- Once/month
- Less than once/month
- Never

7. During the period the UK was under full lockdown (when **outdoor exercise** was **permitted** but **non-essential establishments** e.g. clothes shops, restuarants, pubs etc. were **closed**), how often did you usually go for walks/spend time in the countryside/green spaces?

- At least once/day
- 4-6 times/week
- 2-3 times/week
- Once/week
- Once every two weeks
- Once/month
- Less than once/month
- Never

8. When many lockdown measures were eased (**non-essential** establishments e.g. clothes shops, restuarants, pubs etc. **reopened**), how often did you usually go for

walks/spend time in the countryside/green spaces? **(This may either refer to now or in the past if you are in a part of the UK that has re-entered a stricter lockdown.)**

- ○ At least once/day
- ○ 4-6 times/week
- ○ 2-3 times/week
- ○ Once/week
- ○ Once every two weeks
- ○ Once/month
- ○ Less than once/month
- ○ Never

9. Have you changed where you walk/spend time outside at all since the UK entered lockdown? (select all that apply)

- ☐ Yes, I have been walking closer to home
- ☐ Yes, I have been walking further from home
- ☐ Yes, I have been walking in more remote/rural areas e.g. to avoid other people
- ☐ Yes, I have been walking in more urban areas
- ☐ Yes, I have been exploring a wider range of locations
- ☐ Yes, I have been sticking only to a few places I know well
- ☐ No change

10. When you go out for walks, who do you usually go with? (Select all that apply)

- ☐ On your own
- ☐ With one other adult
- ☐ With a group of adults
- ☐ With children

- [ ] In a group with multiple adults and children

11. When you are walking do you ever stray off the main path/footpaths/bridle paths? (select all that apply)

- [ ] No
- [ ] Occasionally by accident
- [ ] Yes, if it provides a shortcut
- [ ] Yes, so I can explore/access to areas I would otherwise be unable to reach
- [ ] Yes, as long as I am sure the land is not privately owned
- [ ] Yes, so I can interact with wildlife, horses or livestock
- [ ] Yes - Other

11.a. If you selected Yes - Other, please specify:

12. Since lockdown first began, overall have you noticed a difference in the amount of litter you have seen when you are out walking/spending time in the countryside/green spaces?

- ○ I have noticed more litter
- ○ I have noticed less litter
- ○ No change in the amount of litter

Page 4: Use of Green Spaces and Interaction with Wildlife

13. When you go out for walks, do you ever interact with any wildlife in the following ways? (Wildlife refers to any wild animal that is not privately owned) (Select all that apply)

- [ ] I do not usually come across any wildlife
- [ ] I watch/look at wildlife
- [ ] I feed wildlife
- [ ] I physically interact with wildlife e.g. touching/picking up
- [ ] No, I do not interact with wildlife in any way

14. If you have fed wildlife, what kinds of things have you fed it? (If you have fed different things to different animals, please specify what you have fed to each animal)

15. When you go out for walks, do you ever interact with any other animals (e.g. livestock, horses) in the following ways? (This does **not** include dogs being walked by their owners, or any animals owned by you) (select all that apply)

- [ ] I do not usually come across any other animals
- [ ] I watch/look at other animals
- [ ] I feed horses
- [ ] I feed livestock
- [ ] I feed animals other than horses or live stock (please specify)
- [ ] I physically interact with other animals e.g. touching/picking up
- [ ] No, I do not interact with other animals in any way

15.a. If you selected I feed animals other than horses or live stock, please specify:

16. If you have fed other animals, what kind of things have you fed them? (If you have fed different things to different animals, please specify what you have fed to each animal)

17. At any point since the UK entered lockdown and up until now, have you been interacting with animals more regularly than you did before? (select all that apply)

- ☐ I interact with animals less than I did before
- ☐ No Change
- ☐ I watch animals more
- ☐ I physically interact with wildlife more
- ☐ I physically interact with horses more
- ☐ I physically interact with livestock more
- ☐ I feed wildlife more
- ☐ I feed horses more
- ☐ I feed livestock more
- ☐ N/A - I did not interact with animals before and have not at any point throughout lockdown

18. If you have changed the amount you interact with animals whilst walking/spending time outside since lockdown began, what are the reasons for this?

Page 5: Benefits of Accessing Green Spaces

19. If you have been walking more since lockdown occurred, do you feel you have benefitted from this? (Select all that apply)

- [ ] No change
- [ ] Unsure
- [ ] Physical health has benefitted
- [ ] Mental health has benefitted
- [ ] Negative impact on physical health
- [ ] Negative impact on mental health
- [ ] Other
- [ ] N/A

19.a. If you selected Other, please specify:

20. What do you most enjoy about walking/spending time in the countryside/green spaces? (Select all that apply)

- [ ] The exercise
- [ ] The fresh air
- [ ] Having a change of scenery
- [ ] Spending time in nature
- [ ] Interacting with wildlife
- [ ] Interacting with other animals
- [ ] Other

20.a. If you selected Other, please specify:

21. If you would like to add any further comments, please do so here:

You **must** press **FINISH** in order for your responses to be recorded.

Thank you for answering this questionnaire!

Page 6: Further Information

Thank you for taking the time to answer this questionnaire!

Information about government advice on accessing green spaces safely during the COVID-19 pandemic and the Countryside Code can be found below.

Accessing Green Spaces Safely: https://www.gov.uk/guidance/coronavirus-covid-19-advice-on-accessing-green-spaces-safely

Countryside Code Short COVID-19 Version: https://www.gov.uk/government/publications/the-countryside-code/the-countryside-code?fbclid=IwAR0Ki86TEupJTlFFXbpVSdgDtd40yXsq5vEUa0u5W4GImEqpP7dTsZNB7Kg#protect-the-natural-environment

Countryside Code Full Version: https://www.gov.uk/government/publications/the-countryside-code/countryside-code-full-online-version

For SurveyCircle users (www.surveycircle.com): The Survey Code is: 6B4Z-3NQM-P7H2-B1DT
